# Paroxysmal Nocturnal Hemoglobinuria in Pregnancy: A Dilemma in Treatment and Thromboprophylaxis

**DOI:** 10.1155/2017/7289126

**Published:** 2017-09-24

**Authors:** Arpan Patel, Athira Unnikrishnan, Martina Murphy, Robert Egerman, Sarah Wheeler, Ashley Richards, John Wingard

**Affiliations:** ^1^Department of Medicine, Division of Hematology & Oncology, University of Florida, Gainesville, FL, USA; ^2^Department of Obstetrics and Gynecology, University of Florida, Gainesville, FL, USA; ^3^Department of Pharmacology, University of Florida, Gainesville, FL, USA

## Abstract

Paroxysmal nocturnal hemoglobinuria (PNH) is a hematologic disorder characterized by an acquired somatic mutation in the phosphatidylinositol glycan class A gene which leads to a higher risk for increased venous and arterial thrombosis. Current treatment for PNH includes eculizumab. Pregnant patients who have PNH have higher risk for thrombosis and hemorrhage with both pregnancy and their underlying PNH. Treatment frequently poses conundrum. The safety and efficacy of eculizumab during pregnancy and breast feeding have not been extensively studied and contraception has been recommended due to potential for teratogenicity. We present a case of a patient who was safely on both eculizumab and modest prophylactic anticoagulation for 6 weeks post-partum.

## 1. Introduction

Paroxysmal nocturnal hemoglobinuria (PNH) is a complex hematologic disorder characterized by an acquired somatic mutation in the phosphatidylinositol glycan (PIG-A) gene resulting in a deficiency of the glycosyl phosphatidylinositol (GPI) anchored proteins, particularly CD55 and CD59 on the cell membrane [1]. This leads to excessive complement activation resulting in intravascular hemolysis resulting in increased inflammatory cytokines, systemic hemoglobin release, and nitric oxide depletion possibly causing dystonia, severe fatigue, and abdominal pain. PNH patients are at high risk for increased venous and arterial thrombosis and are noted for unusual sites of thrombosis. The complex interactions between the coagulation and complement cascades along with activated platelets may, in part, explain the increased risk of venous thrombosis [2]. PNH is also associated with aplastic anemia and MDS.

Current treatment for PNH includes eculizumab, a monoclonal antibody that blocks terminal complement activation. This results in the reduction in hemolysis and, for many, improvement in the quality of life [3]. Pregnant PNH patients have a higher risk for thrombosis and hemorrhage and frequently pose a treatment conundrum [4]. The safety and efficacy of eculizumab during pregnancy and breast feeding have not been extensively studied. Contraception has been recommended due to its potential for teratogenicity [5]. Another therapeutic dilemma is the role of anticoagulation during pregnancy to mitigate the high risk for a thrombotic disease which can increase during pregnancy and post-partum. We present a case of a patient who was on both eculizumab and modest prophylactic anticoagulation for six weeks post-partum.

## 2. Case Presentation

We present the case of a nulliparous 24-year-old female with no prior past medical history of hereditary conditions predisposing to thrombosis, a body mass index of 25, no family history of thrombosis, and the only acquired and secondary risk of thrombophilia due to PNH, who was referred to our institution for consideration of hematopoietic stem cell transplant. The transplant was offered, and she declined. She was placed on eculizumab for control of her disease and to decrease blood transfusion requirements. Due to potential teratogenicity of eculizumab, she was counseled against becoming pregnant. She tolerated eculizumab treatment well and her transfusion requirements decreased; however, after six months of eculizumab, screening HCG testing revealed she was pregnant. At this time her risk factors for thrombosis were PNH and pregnancy. Due to a confirmed pregnancy, eculizumab was temporarily held for two months. After discussions with the patient regarding the potential risks, eculizumab was restarted in the 10th week of her pregnancy. With the concomitant thrombotic risk conferred by PNH as well as a newly diagnosed pregnancy, thromboprophylaxis was strongly considered. Given a clone size above 50%, anticoagulation with unfractionated heparin was administered antepartum and for six weeks post-partum. A dose of heparin, 5000 units subcutaneously twice daily, was chosen due to her ongoing thrombocytopenia with platelets in the 20,000/mcL. The patient was followed closely on weekly basis to address compliance, monitor blood counts, and evaluate thrombosis and complications of anticoagulation. The patient underwent elective induction of labor at 37 weeks' gestation and delivered a healthy neonate. She remained transfusion independent throughout her pregnancy and experienced no bleeding or thrombotic complications. With regard to her PNH, she experienced no significant change in her granulocyte clone size during her pregnancy.

## 3. Discussion

Paroxysmal nocturnal hemoglobinuria (PNH) is a clonal blood disorder that can present with hemolytic anemia and in some cases smooth muscle dystonia, thrombosis, and bone marrow failure [1]. PNH is classified as de novo or associated with aplastic anemia. It is due to a mutation of the PIG-A gene that ultimately leads to a decrease of anchored proteins, specifically CD55 and CD59 [1]. The loss of these two complement inhibitory proteins renders a patient more susceptible to intravascular and extravascular complement mediated hemolysis. Treatment when symptomatic consists of allogeneic hematopoietic stem cell transplant (HSCT) or eculizumab, a monoclonal antibody that blocks terminal complement activation [1]. For patients with PNH, pregnancy poses a uniquely challenging situation as during pregnancy PNH often worsens, increasing morbidity and mortality for both the mother and the fetus [6].

Eculizumab is a pregnancy class C medication, and animal studies showed a rare complication of retinal dysplasia and umbilical herniation [7]. There are limited data on safety for this specific population of patients. One series looked at 75 pregnant patients on eculizumab and showed that 61 women had no maternal deaths and three fetal deaths were reported (4%) [8]. There were six miscarriages reported during the first trimester (8%) [8]. Twenty cord blood samples were tested for eculizumab, and the drug was detected in seven of the samples [8]. In addition to cord blood samples, ten breast milk samples were tested and were negative [8]. In a series of two pregnant patients treated within nine days of delivery, eculizumab administration to the parturient did not affect complement activity in the newborns [9]. Our patient did not breast-feed her infant. Based on the current literature we chose to continue eculizumab during this patient's pregnancy. The dose and administration frequency of eculizumab may have to be increased during pregnancy should hemolytic parameters deteriorate [10].

Despite the increased risk of thrombosis and death in patients with PNH, there are currently no established guidelines for DVT prophylaxis during pregnancy and post-partum [11]. However, many agree that, in pregnant patients with PNH with a clone size more than 50%, thromboprophylaxis is warranted [11]. In two case series, bleeding complications were noted in around 10% of patients and were noted to be mostly post-partum [7, 11]. In the case series by Sasano et al., both patients received thromboprophylactic doses of heparin during their first pregnancy [11]. Correspondingly, in series by Kelly et al., seven patients were on prophylactic low molecular weight heparin (LMWH), two were on therapeutic doses, and one was on no anticoagulation [8]. Thrombocytopenia is not completely addressed in prior studies, thus complicating the decision-making process [8, 12, 13]. Further, there may be a disparity in the thromboembolic risk between PNH patients from Japan and those from American PNH patients, with the latter group having a higher risk [12]. This further complicates extrapolation of a therapeutic approach from the medical literature. Current, albeit limited, literature supports the safety and efficacy of thromboprophylaxis in pregnant women with PNH due to an increased risk for VTE by virtue of ongoing hemolysis coupled with the hypercoagulable state of pregnancy [6, 10, 14–17]. Many reported patients have been treated with LMWH in either therapeutic, prophylactic, or intermediate doses (REF). Studies and trials have shown that UFH and LMWH are both safe and effective for prophylaxis in patients at high risk of thrombosis [18]. Given our patient's profound thrombocytopenia (platelet counts ranging between 20 and 30,000 during pregnancy), we opted to use UFH 5000 subcutaneously BID in an effort to safely balance risk of bleeding with the need for thromboprophylaxis.

Our patient tolerated her pregnancy well and had no thrombotic complications. At her six-month post-partum follow-up the child had no medical complications. The patient continued eculizumab without grade 3 or 4 side effects and remains minimally transfusion dependent requiring transfusions once every three months as opposed to weekly. This case highlights the difficulty in management of PNH during pregnancy with eculizumab in the setting of severe thrombocytopenia.
